# Adipose Inositol Monophosphate Metabolism Is Associated with Fasting Regimen-Elicited Metabolic Benefits

**DOI:** 10.3390/biom15111514

**Published:** 2025-10-27

**Authors:** Chunqing Wang, Bilian Liu, Xin Yang, Xi Chen, Shuo Wang, Xing Zhang, Meilian Liu

**Affiliations:** 1 Department of Biochemistry and Molecular Biology, University of New Mexico Health Sciences Center, Albuquerque, NM 87131, USA; chuwang@salud.unm.edu (C.W.); xinyang@salud.unm.edu (X.Y.); 2 Autophagy Inflammation and Metabolism Center for Biomedical Research Excellence, University of New Mexico Health Sciences Center, Albuquerque, NM 87131, USA; 3 National Clinical Research Center for Metabolic Diseases, Key Laboratory of Cardiometabolic Medicine of Hunan Province, Metabolic Syndrome Research Center, The Second Xiangya Hospital of Central South University, Changsha 410081, China; liubilian80@csu.edu.cn; 4 The National and Local Joint Engineering Laboratory of Animal Peptide Drug Development, Hunan Normal University, Changsha 410081, China; 202320142849@hunnu.edu.cn (X.C.); 202420142951@hunnu.edu.cn (S.W.); 5 Hunan Provincial Key Laboratory of Animal Intestinal Function and Regulation, College of Life Science, Hunan Normal University, Changsha 410081, China

**Keywords:** intermittent fasting, inositol monophosphate, metabolomics, adipose tissue, obesity

## Abstract

Intermittent fasting (IF) has emerged as a promising strategy for managing obesity and related metabolic disorders. Although metabolic adaptations in adipose tissue during IF are well documented, the specific reprogramming of white adipose tissue (WAT) under prolonged cycles of fasting and refeeding remains incompletely understood. Using mass spectrometry-based approaches, including liquid chromatography (LC) and capillary electrophoresis (CE), we identified a marked increase in inositol monophosphates (InsP1s) in obese adipose tissue following extended IF. Specifically, myo-inositol-1-phosphate and myo-inositol-3-phosphate, which are typically present at low levels in gonadal WAT (gWAT) of diet-induced obese mice, were significantly elevated after 15 cycles of IF. Additionally, extended IF upregulated the expression levels of inositol tetrakisphosphate 1-kinase (ITPK1) and inositol monophosphatase 1 (IMPA1), two key enzymes involved in InsP1 metabolism. These increases coincide with reductions in body weight and fat mass, as well as improved insulin sensitivity. This reprogramming was further supported by enhanced tricarboxylic acid (TCA) cycle activity. Collectively, these findings suggest the inositol monophosphate pathway as a novel mechanism underlying fasting-induced metabolic adaptation in adipose tissue and highlight the potential of these metabolites as biomarkers for obesity and related metabolic conditions.

## 1. Introduction

White adipose tissue (WAT) is traditionally recognized as the body’s primary site where energy storage takes place in the form of triglycerides. However, emerging research reveals that WAT is also a highly dynamic and metabolically active organ, capable of remarkable adaptations in response to nutritional and environmental cues. Among these cues, intermittent fasting (IF), a dietary regimen characterized by alternating periods of fasting and feeding, has gained significant attention for its profound effects on metabolism and overall health.

Intermittent fasting triggers a series of metabolic and cellular adaptations within WAT that go beyond simple fat mobilization. These adaptations include the beigeing of white adipocytes, interorgan communications, increased angiogenesis (formation of new blood vessels), and changes in immune cell populations within the tissue [1,2,3,4,5,6,7,8,9,10,11,12,13]. Such processes enhance the tissue’s capacity for energy expenditure, improve insulin sensitivity, and reduce inflammation. Additionally, IF has been shown to elicit its metabolic benefits by increasing WAT mitochondria protein content and metabolic reprogram, improving glucose tolerance and dyslipidemia in circulation. Metabolic mediators, including gut-derived fermentation byproducts like acetate and lactate, as well as adipocyte-secreted prostaglandins and cytokines such as VEGF, contribute to IF-induced thermogenesis by acting on beige adipocytes or resident macrophages, thereby offering a potential therapeutic pathway [4,5,7,9,14]. Although metabolic reprogramming is increasingly recognized as a key mechanism of adaptation, the specific metabolic mediators underlying the metabolic benefits induced by intermittent fasting (IF) remain largely unidentified.

Inositol phosphates (InsPs) represent a diverse family of signaling molecules essential for cellular communication and metabolic regulation [15,16]. These compounds are synthesized through the phosphorylation of myo-inositol, ranging from simple inositol monophosphate (InsP1) to more complex forms such as inositol hexakisphosphate (InsP6) and inositol pyrophosphates (e.g., InsP7) [17,18]. InsPs are either derived from glucose metabolism or recycled from membrane phospholipids in response to signaling cues. Higher-order InsPs, including InsP6 and InsP7, play pivotal roles in modulating insulin signaling [19,20]. For instance, InsP7 interacts with key metabolic regulators such as Akt kinase, a central component of the insulin pathway, where its production by IP6K1 inhibits Akt activity and thereby influences insulin sensitivity [21]. Beyond insulin signaling, InsPs function as energetic molecules, impacting ATP production, glycolytic flux, lipid metabolism, and mitochondrial function [17,19,22]. Notably, InsPs have been shown to promote the browning of white adipocytes, shifting them toward a thermogenic, energy-expending phenotype. This browning process enhances energy expenditure and mitigates fat accumulation, offering protection against obesity [23,24]. Additionally, 5-InsP7 facilitates adiponectin assembly and secretion by regulating thiol-mediated protein quality control in the endoplasmic reticulum, protecting against ischemia–reperfusion injury [25]. While inositol monophosphate (InsP1) serves as a key intermediate in the biosynthesis and recycling of myo-inositol, its contribution to cellular metabolism, growth, and energy balance, as well as its direct connection to metabolic disease, remains largely unexplored.

By employing mass spectrometry techniques, including liquid chromatography (LC) and capillary electrophoresis (CE), we observed significant modifications of adipose metabolites by extended intermittent fasting under obese conditions, with a significant increase in inositol monophosphates, alongside an upregulation of InsP1 metabolism-related enzymes, ITPK1 and IMPA1. This study suggests a potential mechanism involving inositol monophosphates and their related pathway in the adaptation to extended intermittent fasting, offering promising avenues for combating obesity and metabolic diseases.

## 2. Materials and Methods

Animals C57BL/6 mice (Stock No. 000664) were obtained from Jackson Laboratory, as previously described [14]. For the extended alternate day fasting (ADF) study, 6-week-old mice were fed a 45% high-fat diet (HFD) for 12 weeks, followed by 15 cycles of either ADF or ad libitum feeding with the same diet. In the acute ADF study, 10-week-old male mice underwent 3 cycles of ADF or ad libitum feeding on a standard chow diet. All animals were housed in a pathogen-free barrier facility under a 12 h light/dark cycle, with unrestricted access to food and water. 

Intermittent fasting For extended ADF study, 18-week-old HFD-fed male C57/BL6 mice randomly underwent ad libitum (AL) and alternate day fasting (ADF) started with fasting for 24 h (started at 10 a.m.) following refeeding for 24 h (started at 10 a.m.) as described in our previous study [9]. Mice in the ad libitum (AL) group had unrestricted access to food, whereas those in the alternate day fasting (ADF) group underwent 24 h fasting periods alternated with 24 h periods of free access to food. Mice in ADF group were euthanized 24 h post refeeding. For the acute ADF study, 10-week-old male C57BL/6 mice were randomly assigned to either ad libitum (AL) feeding or alternate day fasting (ADF), which concluded with either a fasting phase (ADF-Fas) or a refeeding phase (ADF-Ref).

DEXA To assess body composition, mice were anesthetized via intraperitoneal injection of Ketamine/Xylazine at a dose of 0.1 mL per 10 g of body weight (containing 10 mg/mL Ketamine and 1 mg/mL Xylazine). Following anesthesia, body weight, fat mass, lean mass, and fat percentage were measured using dual-energy X-ray absorptiometry (DEXA) (GE Medical Systems, Madison, WI, USA), following our published protocol [14].

Glucose and insulin tolerance Diet-induced obese mice underwent alternate day fasting or ad libitum for 15 cycles. The glucose handling was determined using glucose tolerance test and insulin tolerance test as described in our previous study [26].

Metabolomics and lipidomic analyses The assays were performed in collaboration with Human Metabolome Technologies (HMT Metabolomics Technology, Boston, MA, USA). Capillary Electrophoresis-Time-of-Flight Mass Spectrometry (CE-TOFMS) was employed to profile hydrophilic and charged metabolites. Briefly, approximately equal mass adipose tissue samples were homogenized in 50% acetonitrile (*v*/*v*) containing internal standards (10 μM) (HMT, Inc., Tsuruoka, Japan) using a tissue homogenizer (3500 rpm for 60 s, repeated 9 times) (Table 1).

The resulting supernatant (400 µL) was filtered through a 5 kDa cut-off filter (ULTRAFREE-MC-PLHCC, Human Metabolome Technologies, Yamagata, Japan) to eliminate macromolecules. The filtration was then concentrated by centrifugation and resuspended in 25 µL of ultrapure water immediately prior to analysis. Metabolite profiling was conducted using CE-TOFMS in both cationic and anionic modes. Sample dilution was performed to enhance the analytical quality of the CE-MS measurements.

Liquid Chromatography-Time-of-Flight Mass Spectrometry (LC-TOFMS) was utilized to profile hydrophobic and lipid metabolites. Adipose tissue samples were homogenized in 500 µL of 1% formic acid in acetonitrile (*v*/*v*) containing internal standards (10 μM) (HMT, Inc., Tsuruoka, Japan) using a tissue homogenizer (1500 rpm for 120 s, repeated twice). Following the addition of 167 µL of Milli-Q water, the mixture was homogenized again and centrifuged (2300× *g*, 4 °C, 5 min). The supernatant was collected, and the remaining pellet was re-extracted with an additional 500 µL of 1% formic acid in acetonitrile and 167 µL of Milli-Q water. After repeating the homogenization and centrifugation steps, the second supernatant was combined with the first. The pooled supernatant was filtered through a 3 kDa cut-off membrane (NANOSEP 3K OMEGA, PALL Corporation, Ann Arbor, MI, USA) to remove proteins, followed by phospholipid removal using a Hybrid SPE phospholipid column (55261-U, Supelco, Bellefonte, PA, USA). The filtrate was then dried and reconstituted in 200 µL of 50% isopropanol in Milli-Q water (*v*/*v*) immediately prior to analysis. Metabolite profiling was performed in both positive and negative ionization modes using LC-TOFMS. Samples were diluted as needed to enhance the analytical quality of the measurements.

For both CE-TOFMS and LC-TOFMS analyses, detected peaks were annotated using HMT’s standard metabolite library and Known-Unknown peak database. Principal component analysis (PCA) was performed based on the annotated peaks, while hierarchical cluster analysis (HCA) results were visualized as a heatmap.

Hematoxylin and eosin (H&E) staining For H&E staining, adipose and liver tissues from mice in the AL and ADF groups were fixed in 10% formalin for 24 h and subsequently embedded in paraffin. Tissue sections (5 μm thick) were then stained with H&E following standard protocols.

### Data Processing and Analysis

Data Processing: Automated integration software (MasterHands ver. 2.17.1.11) was utilized to extract peaks detected in CE-TOFMS and LC-TOFMS analyses. Peak information included mass-to-charge ratio (*m*/*z*), migration time (MT) for CE, retention time (RT) for LC, and peak area. The peak area was normalized to relative peak area using a defined equation. Peak detection thresholds were determined based on a signal-to-noise ratio (S/N) of 3.

Relative Peak Area = Metabolite Peak Area/(Internal Standard Peak Area × Sample Amount)

Annotation of Peaks: Putative metabolites were identified by matching the detected *m*/*z* values and migration time (MT) or retention time (RT) to entries in HMT’s standard library and Known-Unknown peak database. Matching tolerances were set at ±0.5 min for MT, ±0.3 min for RT, ±10 ppm for CE-TOFMS, and ±25 ppm for LC-TOFMS. When multiple peaks corresponded to the same candidate metabolite, a branch number was assigned to distinguish them.

Mass error (ppm) = (Measured Value-Theoretical Value) × 10^6^/Measured Value

Plotting on Pathway Map: Putative metabolite profiles were visualized on metabolic pathway maps using VANTED (Visualization and Analysis of Networks containing Experimental Data) software. The pathway maps were constructed based on known metabolic pathways present in human cells.

Real-time PCR: The extraction of total RNA from white adipose tissues was performed using the PureLink RNA Mini Kit (Thermo Fisher Scientific, Waltham, MA, USA). The purity and concentration of total RNA were measured using Nano Drop spectrophotometer (Thermo Fisher). 1 μg of total RNA was reverse-transcribed with cDNA kit (AB Applied Biosystems). Real-time PCR amplification was detected using Universal SYBR Green Fast qPCR Mix (ABclonal) on a QuantStudio 3 appliedbiosystems (Thermo Fisher Scientific).

Primer sequences: Inpp4a-F: ACTCCATCGCTAGATCGAAAACC, Inpp4a-R: AGGCAATGCTGCTTAGAAAGAT; Impa1-F: TCCAGAAAGCCCGAGACTTTA, Impa1-R: GACATCAGATCGAACGGTCCA; Impa2-F: AGAGGGAGAGTTGGTGCAG, Impa2-R: GTTTCTGTCACAAGATCGGCA; Itpk1-F: GTGCAGGAAGCGAGGGATAG, Itpk1-R: GGATGACATCGGTCAGCTTGT; Itpka-F: GACTCGGAGGACGATCTGCT, Itpka-R: CTTCTGCCAGTGGCTTTTCTG; Itpkb-F: CGGCGCAGGCTGAATAGTAG, Itpkb-R: ATGCCCACTTTCTGGTTCACC. The relative expression levels of target genes were normalized to 36B4 (36B4-F: GAGGAATCAGATGAGGATATGGGA, 36B4-R: AGCAGGCTGACTTGGTTGC). The relative mRNA expression was calculated as the ratio of each target gene’s expression to that of 36B4.

Statistics Hierarchical cluster analysis (HCA) and principal component analysis (PCA) were conducted using statistical software developed by HMT for metabolomic and lipidomic studies. Statistical comparisons between two groups were performed using a two-tailed Student’s *t*-test or Welch’s *t*-test as described in the figure legend. In all cases. A *p* value of <0.05 was considered statistically significant (* <0.05, ** <0.01, *** <0.001).

## 3. Results

### 3.1. Prolonged Alternate Day Fasting Reprograms Metabolism in Visceral Fat

**Figure 1:**
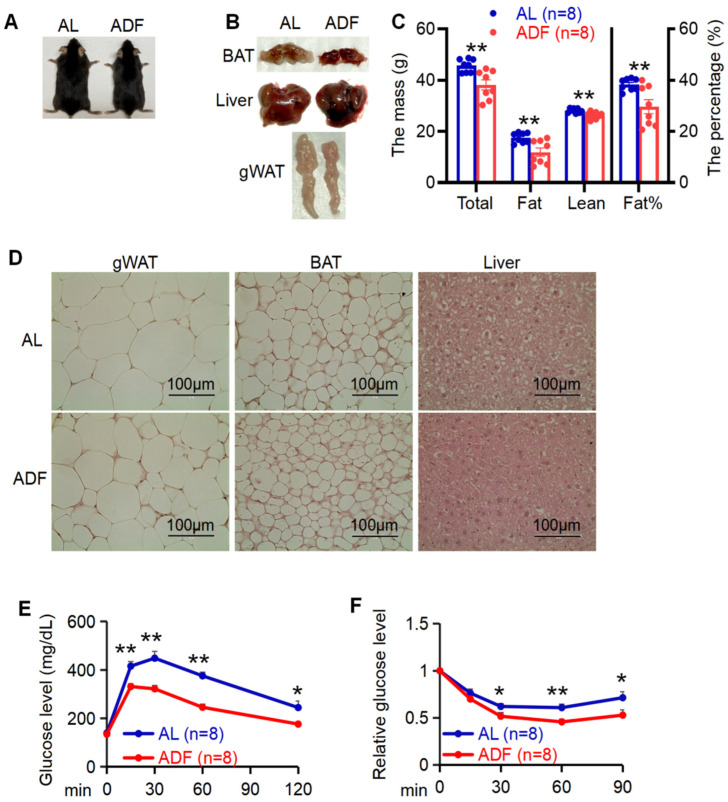
Prolonged alternate day fasting significantly improves systemic metabolism. (**A**): Representative images of diet-induced obese mice with or without alternate day fasting (ADF) for 15 cycles. (**B**). Tissue images for gWAT, BAT, and liver in two groups ad libitum (AL) and ADF. (**C**). 15 cycle-ADF led to body mass loss and fat reduction. Body composition was analyzed by DEXA scanning. (**D**) Histological analysis of gWAT, BAT, and liver in AL and ADF mice. Glucose tolerance (**E**) and insulin tolerance (**F**) were improved by 15 cycle-ADF. Values in (**C**,**E**,**F**) are expressed as mean ± SEM. *t*-test was utilized to analyze the data for (**C**) and unpaired two-tailed Student’s *t*-test was for (**E**,**F**). * *p* < 0.05 and ** *p* < 0.01.

Intermittent fasting (IF) has garnered growing attention as an effective strategy for promoting weight loss and enhancing insulin sensitivity [1,2,3,4,5,7,9]. However, how various fat depots adapt to extended IF remains incompletely understood. To this end, at the age of 6 week, C57BL/6 mice were fed with 45% HFD for 12 weeks followed with 15 cycles of periodic fasting and refeeding (ADF) or ad libitum (AL). The anti-obesity effects of IF were evident as indicated by body mass loss, visceral fat reduction and improved glucose and insulin tolerance between ADF and AL groups (Figure 1A–E).

**Figure 2:**
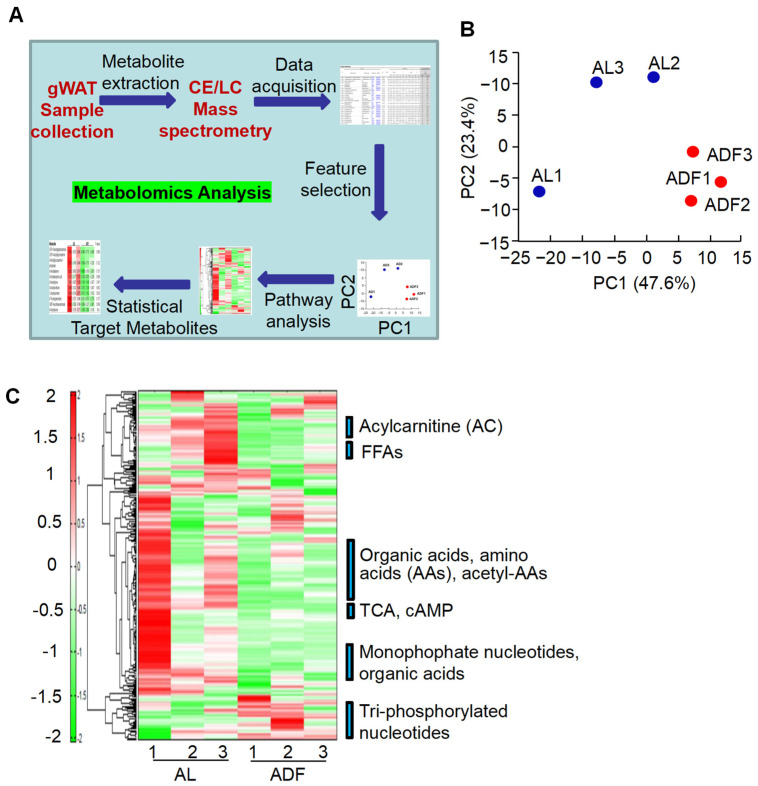
Prolonged alternate day fasting reprograms metabolism in visceral fat. (**A**). Workflow of adipose tissue metabolomic analysis. (**B**). Principal component analysis (PCA) of metabolomics and lipidomics results using the detected peaks, PC1, the first principal component; PC2, second principal component. The numbers in parentheses indicate contribution rates, while the plot labels correspond to sample names. (**C**). Heatmap of adipose tissue metabolites with hierarchical cluster analysis (HCA) in gWAT from ADF and AL groups.

To further explore adipose metabolic effects elicited by IF, we performed lipidomic and metabolomic analyses of gWAT using CE-TOFMS and LC-TOFMS in two modes for cationic and anionic metabolites (Figure 2A). CE-TOFMS analysis targeted 359 metabolites, including 212 in cation mode and 147 in anion mode. LC-TOFMS analysis covered 165 metabolites, with 93 detected in positive mode and 72 in negative mode. From these analyses, 231 metabolites were identified using CE-TOFMS (121 in cation mode and 110 in anion mode), and 80 metabolites were detected using LC-TOFMS (54 in positive mode and 26 in negative mode). Based on metabolite profiling, gWAT exhibited distinct metabolite profiles between ADF and AL groups, which were well separated except for one sample of AL group (Figure 2B).

In general, there were several significant alterations in gWAT induced by extended IF (Figure 2C): (1) Increase in inositol monophosphates (InsP1s) and triphosphate nucleotides in ADF group; (2) the metabolic reprogram is further supported by the refeeding-induced decline in acylcarnitine (AC) persists when intermittent fasting is extended; (3) an activation of TCA cycle accompanied by a reduction in ethanolamine phosphate in ADF group, and (4) gWAT exhibited a decrease in obesity-associated metabolites, including 2-hydroxyglutaric acid, sphingosine, and cysteine glutathione disulfide, after IF treatment.

### 3.2. Alternate-Day Fasting Induces InsP1 Accumulation and Promotes Metabolic Flux Through the TCA Cycle in Visceral Adipose Tissue

**Figure 3:**
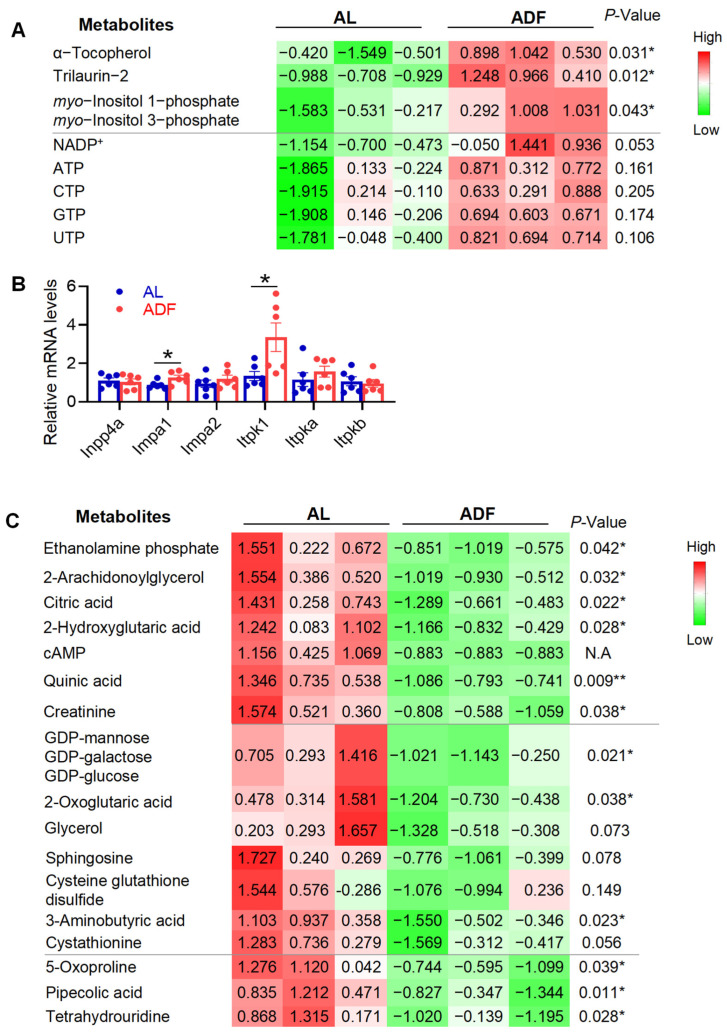
Prolonged alternate-day fasting increases InsP1 levels and stimulates TCA cycle activity in visceral fat. The table summarized the rest of detected and differentially expressed metabolites within gWAT during 15 cycles of ADF in obesity. (**A**). The quantified data for upregulated metabolites in gWAT. (**B**). mRNA levels of those enzymes for InsP1 metabolism. (**C**). Quantified data for downregulated metabolites in gWAT. The *p*-value is computed by Welch’s *t*-test. * *p* < 0.05 and ** *p* < 0.01.

Inositol phosphates (InsP) serve as key signals that modulate cellular metabolism, growth, and energy balance [15,16]. However, the implication of InsP1 in metabolic diseases is unclear. Supporting the notion that extended periodic fasting and refeeding enhances energy metabolism in visceral fat, two InsP1s, myo-inositol 1-phosphate (Ins(1)P1) and myo-inositol 3-phosphate (Ins(3)P1), were significantly elevated following extended alternate-day fasting (ADF) (Figure 3A).Additionally, two fatty acid derivatives were significantly elevated in response to extended ADF: α-tocopherol, the most common form of vitamin E and a potent antioxidant [27], and trilaurin-2, a specific isomer of trilaurin where the lauric acid is confirmed to be on the sn-2 position of glycerol (Figure 3A). Supporting this, extended IF upregulated the expression levels of inositol tetrakisphosphate 1-kinase (ITPK1) and inositol monophosphatase 1 (IMPA1), two key enzymes involved in InsP1 metabolism, but not inositol polyphosphate-4-phosphatase (INPP4a), in gWAT (Figure 3B). Along this line, we observed activation of the tricarboxylic acid (TCA) cycle, as indicated by decreased levels of citric acid and 2-oxoglutarate, suggesting accelerated cycle turnover (Figure 3C). Since samples were collected during the refeeding phase, the post-fasting rebound was associated with a significant reduction in metabolites linked to lipolysis, including 2-arachidonoylglycerol and ethanolamine phosphate (Figure 3C).

Additionally, two fatty acid derivatives were significantly elevated in response to extended ADF: α-tocopherol, the most common form of vitamin E and a potent antioxidant [27], and trilaurin-2, a specific isomer of trilaurin where the lauric acid is confirmed to be on the sn-2 position of glycerol (Figure 3A). Furthermore, extended ADF appears to promote a healthier metabolic profile in visceral fat, as evidenced by reduced levels of hypoxia-associated metabolites, such as 2-hydroxyglutaric acid, sphingosine, and cysteine-glutathione disulfide (Figure 3C). Several other metabolites were significantly decreased in the ADF group compared to ad libitum-fed controls, including quinic acid, creatinine, GDP-mannose (or GDP-galactose/GDP-glucose), 5-oxoproline, pipecolic acid, and tetrahydrouridine (Figure 3C). Collectively, these findings suggest that extended alternate-day fasting may enhance the efficiency of energy metabolism in adipose tissue and support a more favorable metabolic state, though further investigation is warranted.

### 3.3. InsP1s and Triphosphate Nucleotides Exhibit Gradual Modulation in Adipose Tissue in Response to the Fasting Regimen

**Figure 4:**
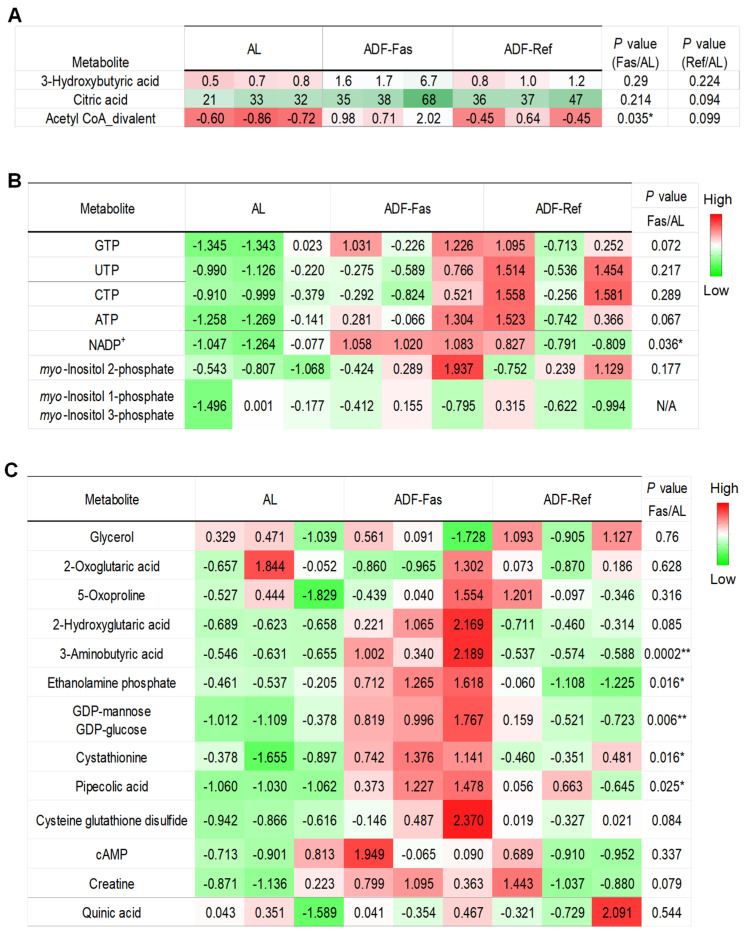
Gradual modulation of InsP1s and triphosphate nucleotides in adipose tissue in response to the fasting regimen. The table summarized the rest of detected and differentially expressed metabolites within gWAT during 3 cycles of ADF under chow diet conditions. The data were quantified for 3-hydroxybutyric acid, citric acid, and acetyl-coA divalent in (**A**), InsP1s and triphosphate nucleotides in (**B**), and other metabolites in (**C**). The *p*-value is computed by Welch’s *t*-test. * *p* < 0.05 and ** *p* < 0.01.

To assess whether metabolic alterations emerge during the early stages of alternate-day fasting (ADF), we analyzed select metabolites across three cycles of ADF, capturing both fasting (Fas) and refeeding (Ref) phases in 3-month-old male mice maintained on a standard chow diet. As expected, 3-hydroxybutyric acid, a key ketone body, was elevated during fasting and returned to baseline levels upon refeeding (Figure 4A). Although myo-inositol 2-phosphate (Ins(2)P1) was captured under acute ADF conditions, its levels were not significantly affected by acute ADF like Ins(1)P1 and Ins(3)P1, suggesting a gradual elevation of InsP1s during intermittent fasting. NADP^+^ was significantly elevated during the fasting phase but not during refeeding (Figure 4B). In contrast to findings from the extended ADF study, the downregulation of TCA cycle intermediates, cAMP, and hypoxia-associated metabolites observed in prolonged ADF was absent following short-term treatment, which was even elevated significantly on fasting state (Figure 4C). Both α-tocopherol and trilaurin-2 were not detected in this acute setting. Collectively, these results suggest that the metabolic reprogramming in adipose tissue is very dynamic during intermittent fasting, potentially via InsP1-related biochemical pathways, including inositol recycling, signaling, and the synthesis of higher-order inositol phosphates.

### 3.4. Extended Alternate Day Fasting Decreases Acylcarnitine (ACs) Levels in Visceral Fat

**Figure 5:**
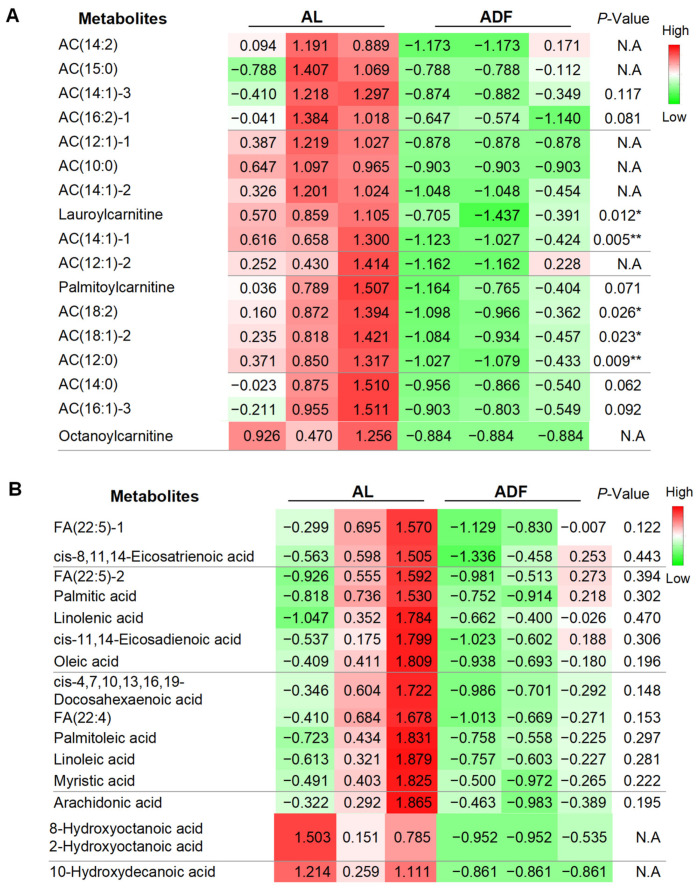
Extended alternate day fasting decreases acylcarnitine (ACs) levels in visceral fat. The table summarized detected and differentially expressed metabolites within gWAT. The data were quantified for individual acylcarnitine (**A**) and free fatty acids (FFAs) (**B**). The *p*-value is computed by Welch’s *t*-test. * *p* < 0.05 and ** *p* < 0.01.

Consistent with findings from acute intermittent fasting (IF) (Zhang X et al., under revision), refeeding during extended IF reduced a broad range of acylcarnitine (AC) levels, spanning carbon 12 to carbon 19 (Figure 5A). The most pronounced reductions were observed in lauroylcarnitine, AC(12:0), AC(14:1)-4, AC(18:2), and AC(18:1)-2 (Figure 5A). In contrast, the extent of reduction in other ACs was smaller, likely due to low baseline levels, non-availability (N.A.), or variability (Figure 5A). These results suggest that prolonged alternate-day fasting (ADF) may stimulate fatty acid oxidation in visceral fat, a cellular process that metabolizes lipids for energy. These results suggest that refeeding-induced reduction in AC and FFA persist when alternate day fasting is extended, albeit to a lesser extent. Compared to lipid profile data from acute fasting and refeeding (Zhang X et al., under revision), our present study also suggests that extended ADF may diminish the metabolic distinction between fasting and refeeding states, as refeeding consistently leads to a marked decrease in both AC and FFA levels.

## 4. Discussion

Dietary interventions such as calorie restriction and fasting regimens can reverse metabolic dysfunction by enhancing energy consumption (fatty acid oxidation) and metabolic reprogramming at molecular levels [4,5,7,28]. However, an important question remains: can biomarkers of metabolic dysregulation also serve as indicators of metabolic improvement? In our present study, we observed that extended alternate day fasting (ADF) appears to promote the accumulation and metabolism of inositol monophosphates (InsP1s), particularly myo-inositol 1 phosphate (Ins(1)P1) and myo-inositol 3 phosphate (Ins(3)P1). Given inositol phosphates are well recognized regulator for cell metabolism, growth, and energy balance, in obese adipose tissue, these findings highlight the potential of InsP1s pathway as a novel biomarker for obesity, mediating fasting regimen-induced metabolic benefits. Further investigations are necessary to clarify the implications of InsP1s in metabolic health and disease.

Inositols, particularly myo-inositol and inositol phosphates (InsPs), have garnered significant attention for their roles in metabolic health and disease. These molecules are implicated in energy homeostasis, antioxidant and anti-inflammatory responses, and even function as neurotransmitters [29], with emerging evidence supporting their anti-obesity properties [30]. Soluble inositol phosphates consist of an inositol ring phosphorylated at one or more positions in various combinations. Although this group has a broader diversity than lipid-based inositol derivatives, only a small subset has been extensively studied and recognized for its importance in metabolic signaling. Among these, higher-order InsPs such as InsP6 and InsP7 act as key second messengers, playing critical roles in regulating cellular metabolism [19,20]. In contrast, inositol monophosphate (InsP1) has been poorly characterized in the context of metabolic health. Our current study demonstrates that Ins(1)P1 and Ins(3)P1 may serve as hallmarks of obesity, with their elevation potentially reflecting metabolic improvements induced by intermittent fasting (Figure 3 and Figure 4). Interestingly, Ins(2)PI but not Ins(1)P1 and Ins(3)P1 respond to acute ADF treatment, reflective of dynamics and selectivity of InsP1 alterations in response to fasting regimen. Ins(3)P1 can originate from glucose-6-phosphate, while Ins(1)P1 are derived from sphingolipids [22]. However, it remains unclear whether glucose-6-phophate and sphingolipids contribute to ADF-induced accumulation of InsP1s in adipose tissue. Moreover, the biological functions of InsP1 in adipose tissue metabolic adaptation require further investigation to fully understand its role in fasting-mediated metabolic reprogramming.

Inositol phosphates are closely linked to the phosphatidylinositol signaling pathways, including PtdIns(4,5)P_2_ and PtdIns(3,4,5)P_3_, which give rise to inositol polyphosphates (IPs) and inositol pyrophosphates (PP-IPs)—newly recognized players in metabolic control [17]. PtdIns(4,5)P_2_ can release I(1,4,5)P_3_, which is either dephosphorylated to myo-inositol for recycling into PtdIns or converted into higher-order IPs via kinase-mediated reactions [31]. For recycling, IP_3_ is converted to myo-inositol through the action of inositol polyphosphate-4-phosphatase (INPP4) and inositol polyphosphate-5-phosphatase (INPP5) [31]. Consequently, InsP1 is not only a product of INPP4 activity but also has two potential fates: Dephosphorylation to myo-inositol via inositol monophosphatase and Conversion into higher-order IPs via inositol monophosphate 1-kinase (ITPK1) [22]. We observed the upregulation of ITPK1 and IMPA1 but not inositol polyphosphate-4-phosphatase (INPP4) and IMPA2, suggesting the activation of myo-inositol recycling and InsP1 metabolism. Further studies will be needed to clarify whether InsP1s are key substrates for ITPK1 in gWAT, mediating IF-induced InsP1 production. If so, which high-order InsPs are involved in fasting regimen-induced improvement of metabolic homeostasis?

The elevation of InsP1 in adipose tissue also suggests its potential translational and clinical relevance. Further efforts are needed to determine whether fasting regimen alters inositol levels in human adipose tissue and blood. Of note, IMPA1 is the enzyme responsible for dephosphorylating Ins(1)P1 to regenerate free inositol, a crucial step in inositol recycling and signaling. The observed increase in IMPA1 expression in the context of elevated Ins(1)P1 levels suggests an adaptive response by the pathway to restore homeostasis by accelerating the conversion of accumulated Ins(1)P1 back to inositol. On the other hand, how extended intermittent fasting affects inositol phosphate (InsP) metabolism remains an intriguing question. Hormonal fluctuations and signaling pathways likely play a significant role in this process; however, the precise mechanisms underlying these changes require further investigation and clarification in future studies.

The observed increase in InsP1s may be associated with shifts in lipid metabolism, including the activation of fatty acid oxidation, enhanced tricarboxylic acid (TCA) cycle activity, and ultimately ATP production under extended intermittent fasting (IF) conditions. Although direct evidence linking InsP1 to TCA cycle regulation is currently lacking, both InsP1 and other inositol phosphates are known to influence cellular energy metabolism, suggesting a potential modulatory role. Given that InsP3 functions as a key second messenger in processes such as lipogenesis, lipolysis, and fatty acid oxidation [18], it is plausible that InsP1 may similarly impact lipid-related metabolic pathways. One critical intermediate in lipid metabolism is acylcarnitine, which facilitates the transport of fatty-acyl-CoA into mitochondria for β-oxidation (Figure 5A). Reduced levels of acylcarnitine may reflect diminished lipid storage, likely due to decreased availability of fatty acids for triglyceride synthesis during fasting. This reduction may also be influenced by dietary fatty acid uptake and rapid turnover (Figure 1B,C). Consistent with enhanced lipid utilization, accelerated TCA cycle turnover contributes to elevated ATP production, supporting energy demands during fasting. Fatty acid oxidation becomes a primary energy source in response to metabolic stressors such as fasting. The observed decline in fatty acyl-CoA levels may indicate adipose tissue adaptation to prolonged fasting, contributing to metabolic homeostasis. Interestingly, the reduction in free fatty acid levels persists even during refeeding, albeit to a lesser extent than during acute fasting and refeeding phases (Zhang X et al., under revision). This trend suggests a blunted metabolic contrast between fasting and refeeding states following extended metabolic adaptation, highlighting the dynamic nature of lipid metabolism in response to sustained dietary interventions. The functional connections between InsP1s and other metabolically altered pathways, including fatty acid oxidation, the TCA cycle, and ATP production, also warrant further investigation to clarify their roles in adipose tissue adaptation and systemic energy metabolism.

## 5. Conclusions

In conclusion, our study identified relatively low levels of inositol monophosphates (InsP1s), specifically myo-inositol 1-phosphate (Ins(1)P1) and myo-inositol 3-phosphate (Ins(3)P1), in obese white adipose tissue. The marked increase in these metabolites and their metabolism during prolonged intermittent fasting was associated with metabolic reprogramming in visceral fat and enhanced metabolic function. Supporting this, the extended fasting regimen enhances myo-inositol recycling and InsP1 metabolism. These findings suggest that inositol monophosphate pathway may serve as a novel biomarker for obesity and the metabolic benefits conferred by fasting regimens, offering new mechanistic insights into the physiology of fasting.

## Figures and Tables

**Table 1:** Sample Information.

Name	Amount (mg) ‡ (CE-TOFMS)	Amount (mg) (LC-TOFMS)	Group	Dilution (Cation) §	Dilution (Anion) §	Dilution (Positive) §	Dilution (Negative) §
A1	142.8	48.1		1	2	1	1
A2	145.7	49.5	AL	1	2	1	1
A3	142.2	47.9		1	2	1	1
B1	147.4	50.0		1	2	1	1
B2	146.6	48.1	ADF	1	2	1	1
B3	146.3	48.3		1	2	1	1

‡ Amount of samples was measured in HMT. § Dilution factors for Measurement.

## Data Availability

The original contributions presented in this study are included in the article. Further inquiries can be directed to the corresponding author.

## Abbreviations

The abbreviations used are: HFD: high fat diet; AL: ad libitum; ADF: alternate day fasting; gWAT, gonadal WAT; BAT: brown adipose tissue; InsP1: inositol monophosphate; InsP: inositol phosphate; Ins(1)P1: myo-inositol 1-phosphate; Ins(12)P1: myo-inositol 2-phosphate; Ins(3)P1: myo-inositol 3-phosphate; ITPK1: inositol monophosphate 1-kinase; IMPA1: inositol monophosphatase 1; IMPA2: inositol monophosphatase 2; INPP4, inositol polyphosphate-4-phosphatase: TCA: tricarboxylic acid.
